# “The Therapy of Elimination First” for Early Acute Mastitis: A Systematic Review and Meta-Analysis

**DOI:** 10.1155/2018/8059256

**Published:** 2018-05-02

**Authors:** Ying Zhang, Xiaoying Sun, Kexin Li, Xiaomin Wang, Lijun Cai, Xin Li, Min Zhou

**Affiliations:** ^1^Department of Dermatology, Yueyang Hospital of Integrated Traditional Chinese and Western Medicine, Shanghai University of Traditional Chinese Medicine, Shanghai 200437, China; ^2^Institute of Dermatology, Shanghai Academy of Traditional Chinese Medicine, Shanghai 201203, China

## Abstract

We evaluated the effectiveness of “the therapy of elimination first” in early acute mastitis, using four databases (CNKI, Wanfang, Embase, and PubMed). The study incorporated 2508 patients from 16 randomized controlled trials (RCTs). Included trials used Chinese oral medicine and applied the principle of “Eliminating Therapy” for the early treatment of acute mastitis, with simple antibiotic treatment as a control group. Meta-analysis showed significant differences between the overall effectiveness of oral Chinese medicine using Eliminating Therapy (OCM-ET) and western medicine using antibiotics (WM-A) (odds ratio [OR] = 4.43, 95% confidence interval [CI] = 3.21–6.12, *Z* = 9.04, and *P* < 0.00001). Analysis of subgroups based on the use of classic or self-made preparations of the medicines showed smaller statistical heterogeneity among the different subgroups (*P* > 0.05, *I*2 ≤ 50%). The OCM-ET group showed significantly shorter pain relief times [mean difference (MD) = −3.08, 95% CI = (−5.90, −0.26), and *P* = 0.03] and cure times [MD = −6.27, 95% CI = (−9.68, −2.85), and *P* = 0.0003] than did the WM-A group. Our findings suggest that OCM-ET can shorten the duration of pain and improve cure time in early acute mastitis patients, with fewer adverse reactions. However, RCTs of higher quality with larger sample sizes are required to confirm these findings.

## 1. Introduction

Acute mastitis is an acute purulent disease of the breast, which is commonly observed in uniparous lactating women, usually at one month postpartum. The incidence of mastitis in China is 18.6% [1]. In traditional Chinese medicine (TCM), acute mastitis is classified as a “Mammary Abscess.” The two principle causes of mastitis are milk stasis and infection. Milk stasis is usually the primary cause [2, 3] and may or may not be accompanied by, or progress to, infection. Early massage and suckling are the keys to avoiding abscess [4]. Acute mastitis is characterized by systemic symptoms such as breast lumps, redness, swelling, heat, and pain. Based on its course, the disease is divided into three stages: the initial (stagnant) stage, the pus formation stage, and the last (restoration) stage [5]. A preliminary study [6] counted leukocytes and bacteria in milk from breasts with clinical signs of mastitis and proposed the following classification: milk stasis, noninfectious inflammation (or noninfectious mastitis), and infectious mastitis. Modern medicine promotes the use of antibiotics in the initial stage, which is effective for acute mastitis caused by bacteria [7]. However, in most cases, acute mastitis is not caused by bacterial infection, and using antibiotics in these situations is therefore not the best choice [8]. It has been confirmed that it is possible to be certain, from clinical signs alone, whether or not infection is present [6]. In some patients, the systemic symptoms are controlled by antibiotics, but in others the local breast lump continues to fester and the systemic symptoms worsen [9]. In TCM, there are various treatments for acute mastitis in the stagnant stage, which are mainly divided into two categories: internal treatments and external treatments. External treatments include the following: massage manipulation, external application of Chinese medicine, acupuncture, moxibustion, cupping, scrapping, and physiotherapy [10]. TCM has certain advantages, such as simpler methodology, lower costs, safety, and rapid effects [11], and is considered the first-choice treatment for acute mastitis in China [12].

In modern times, despite abundant medical resources and increased awareness of maternal hygiene, the incidence rate of acute mastitis is still high. The underlying causes for this include stress, a surplus of nutrients during pregnancy, overreliance on breast pumps, and a lack of breastfeeding experience [13]. Several clinical trials have demonstrated the effectiveness and advantages of TCM for acute mastitis. Although there is abundant literature on the use of Chinese medicine for acute mastitis, its quality and curative effects have not been systematically evaluated. Therefore, a comprehensive study of the existing literature is needed for a better understanding of its application in the treatment of acute mastitis. This study applies the principle of evidence-based medicine and evaluates the clinical effects reported in the standard literature objectively, credibly, and systematically. We have attempted to find evidence for the curative effects and advantages of Chinese medicine for early acute mastitis so as to promote its clinical application.

## 2. Materials and Methods

### 2.1. Data Sources and Searches

To identify relevant randomized clinical trials (RCTs), two reviewers (Ying Zhang and Lijun Cai) systematically searched the China National Knowledge Infrastructure database (CNKI), Wanfang Data Knowledge Service Platform, Excerpta Medica dataBASE (EMBASE), and PubMed, using the search terms “acute mastitis,” “mammary abscess,” “milk congestion,” “traditional Chinese medicine (TCM),” “traditional Chinese herb,” and “herbal medicine.” Articles in English and Chinese published between January 1998 and December 2016 were included in this study. Moreover, the references to all the selected publications and reviews were manually searched for further relevant articles, and a total of 1036 articles were finally included.

### 2.2. Study Selection

The publications included in the study were based on the following inclusion criteria: (1) RCTs, irrespective of whether or not blinding was adopted, (2) studies whose original data should have been published in a public document, (3) subjects of the study who must be women with early stage acute mastitis fulfilling the diagnostic criteria, and (4) experimental groups that should have received oral Chinese medicine using Eliminating Therapy (OCM-ET), while control groups should have received western medicine using antibiotics (WM-A), (5) the index of therapeutic evaluation was normalized, and (6) the cases of experimental groups and control groups were clear and statistical analysis was performed. The exclusion criteria included the following: (1) reviews, experience summaries, case reports, studies involving animals, or a theoretical exploration; (2) the study object which did not fulfill the explicit diagnostic criteria; (3) studies that were not randomized or were not RCTs; (4) lack of control groups or lack of comparable groups; (5) the report which was included in secondary published papers or cited literature.

### 2.3. Data Extraction

Two reviewers (Xiaomin Wang and Kexin Li) extracted the data independently using a predefined data extraction form. Disagreements were resolved by consensus or discussion with a third reviewer (Min Zhou). The data extracted included the first author name, location, baseline characteristics, study characteristics (i.e., year, duration of treatment), participant characteristics (i.e., mean age, sample size, and staging of acute mastitis), drugs used for treatment of the experimental and control groups, suppliers of the TCM, therapeutic principle of the TCM, measured outcomes, evaluation methods, adverse reactions (ADs) of the experimental group, and followup details. For studies with insufficient information, the reviewers contacted the primary authors, when possible, to acquire and verify the data. The abovementioned information was summarized, and the two reviewers crosschecked it.

### 2.4. Methodological Quality Evaluation

The risk of bias in each study was assessed by two authors (Xiaoying Sun and Xin Li) independently using the Cochrane Risk of Bias tool for reference [14]. Disagreements were resolved either by consensus or by a third reviewer (Min Zhou). Evaluation included (1) random sequence generation, (2) allocation concealment, (3) blinding of participants and personnel, (4) blinding of outcome assessment, (5) incomplete outcome data, (6) selective reporting, and (7) other biases such as whether the baseline was balanced, whether there was fraud, or whether there was benefit. The results of risk assessment were categorized as low risk, unclear risk, or high risk.

### 2.5. Data Synthesis and Analyses

All statistical analyses were performed using the Review Manager 5.2 software (Cochrane Community, London, United Kingdom). We compared the final results to assess the differences between experimental and control groups. Cochrane's *χ*^2^ and *I*^2^ tests were used to assess the degree of heterogeneity between studies. Considerable heterogeneity was revealed by *P* values less than 0.10, or *I*^2^ values above 50%, in the *χ*^2^ and *I*^2^ tests, respectively. In this case, a random-effects model was used in order to compute the global odds ratio (OR) and mean difference (MD). Fixed-effect models were used for studies with *P* values greater than 0.05 or *I*^2^ less than 50%, or when the intrastudy heterogeneity was not substantial. Clinical heterogeneity was assessed by reviewing the differences in the distributions of participants' characteristics among the different trials (i.e., age, gender, and duration of disorder).

## 3. Results

### 3.1. Study Selection

**Figure 1:**
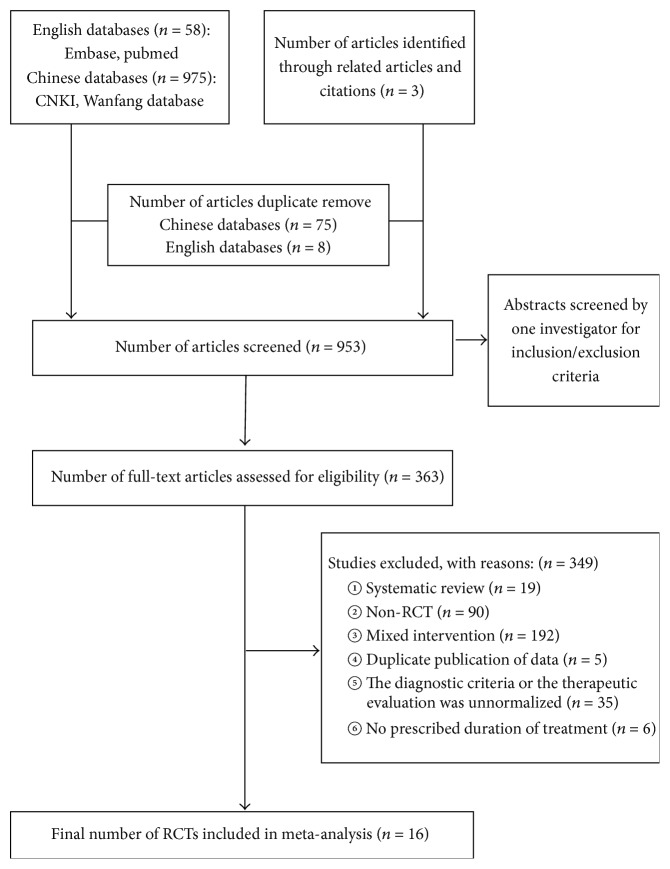
Summary of the literature identification and selection process. CNKI indicates the Chinese National Knowledge Infrastructure database; Wanfang database; Wanfang Data Knowledge Service Platform; Embase, Excerpta Medica dataBASE; RCT: randomized clinical trials.

The preliminary search yielded 1036 studies, of which 83 repeat articles and others such as experience summaries, case reports, animal studies, and theoretical explorations were deleted. The full texts of 363 potentially relevant studies were reviewed to confirm their eligibility. Of these, 19 systematic reviews, 90 non-RCT studies, 192 reports on treatments with mixed interventions, 5 reports with duplicate publications of data, 35 studies that did not meet the diagnostic or efficacy evaluation criteria, and 6 studies with no prescribed duration of treatment were all excluded, finally leaving 16 trials that met the inclusion criteria (Figure 1).

### 3.2. Study Characteristics

All 16 trials included in this study were published in Chinese. A total of 2508 patients participated in these trials, with 1322 and 1186 in the experimental and control groups, respectively. The sample sizes of these trials ranged from 20 to 600. Only one out of these 16 trials reported adverse events in the experimental group [15], and another one reported patient followup [16]. While all 16 studies used Chinese herbal decoctions for the experimental groups, 6 of them used classic preparations including GuaLouNiuBang decoctions [17–19], a Xiaodu decoction [20], and YangHe decoctions [21, 22], and the remaining 10 used self-made preparations [15, 16, 23–30]. The control groups in all the studies were treated with antibiotics, 8 with penicillin [15, 19, 21, 23–25, 28, 29], and the others with cephalosporins [16–18, 20, 22, 26, 27, 30]. All 16 studies used “Eliminating Therapy,” including heat-clearing therapy [15, 17–20, 23, 25–30], warming through therapy [21, 22], and harmonizing ying therapy [16, 24] (Tables 1 and 2).

### 3.3. Risk of Bias Assessment

**Figure 2:**
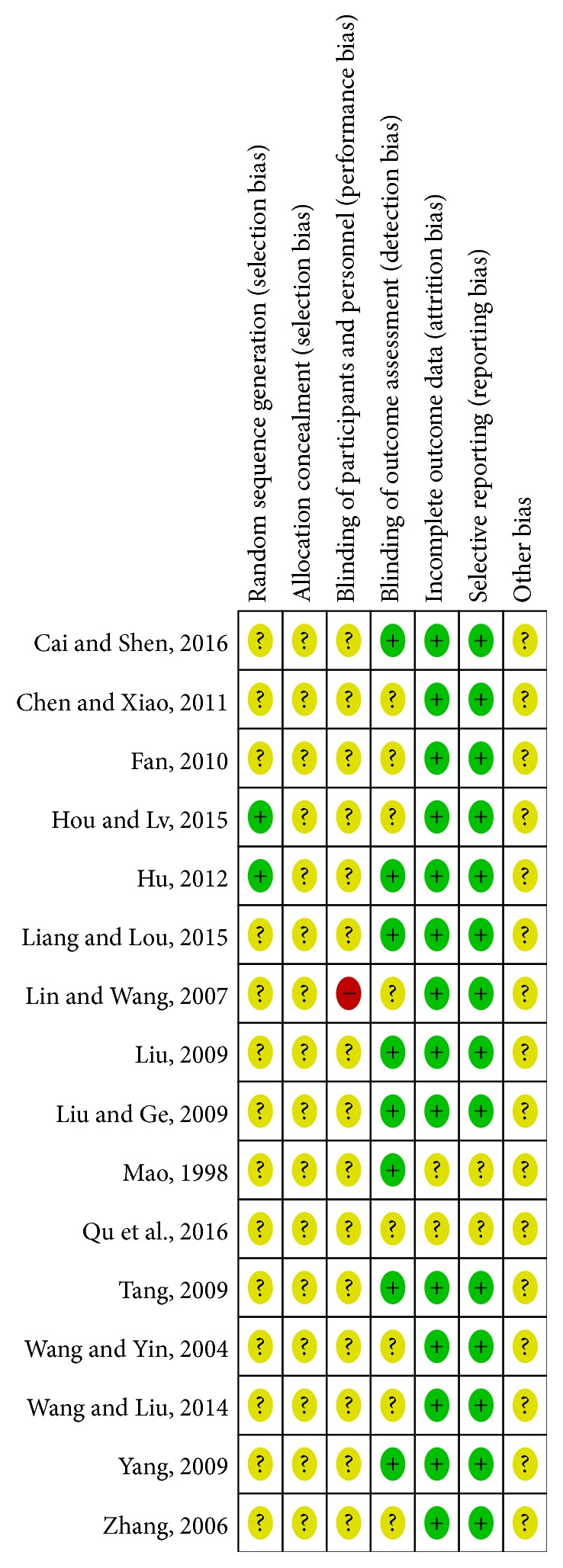
Risk of bias graph.

**Figure 3:**
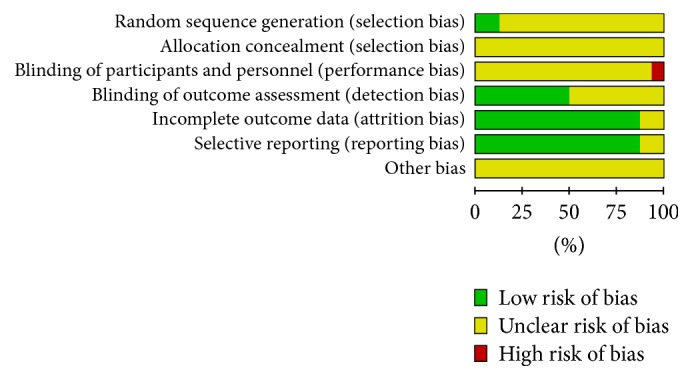
Risk of bias summary.

The methodological quality evaluation categorized all the included trials as inadequate (Figure 2). Although all these trials reported randomization, only two adequately described the randomization method: one with a random number table [28] and the other divided by the envelope method [18]. Moreover, none of the studies reported information such as allocation concealment, blinding of participants and personnel, or blinding of outcome assessment. Eight of the studies were less likely to be affected by the lack of blinding [16, 19–23, 27, 28]. Most of the relevant trials adequately addressed incomplete outcome data and selective reporting, in addition to not reporting such situations [15, 22]. Although no other biases were found in these trials, considering their poor methodological quality, we decided to assign an unclear risk of bias to all the included trials. The components of each risk entry are shown in Figure 3.

### 3.4. Primary Outcomes

**Figure 4:**
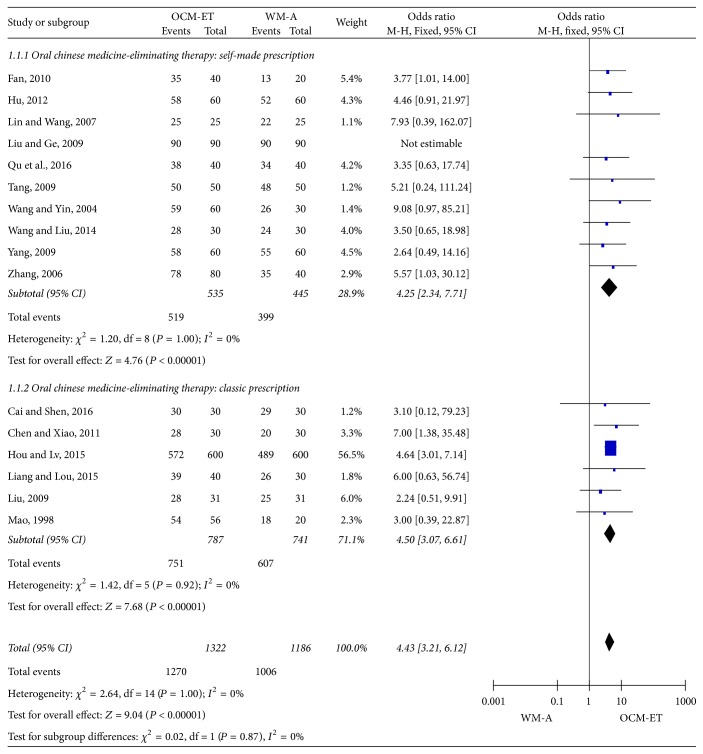
Meta-analysis of the total effectiveness rate against acute mastitis of oral Chinese medicine-Eliminating Therapy (OCM-ET) versus western medicine antibiotics (WM-A) based on whether or not a classic prescription was used. CI indicates confidence interval.

Total effectiveness rates of OCM-ET versus WM-A: the experimental and control groups received OCM-ET and WM-A, respectively. Some subjects from each of the two groups also received basic intervention strategies, including hot compresses [17, 20, 26] and hand milking [19, 30] as a second-line treatment. Pooling of the results from these trials showed a significant difference in the total effectiveness rate between the OCM-ET and WM-A groups (OR = 4.43, 95% confidence interval [CI] = 3.21–6.12, *Z* = 9.04, and *P* < 0.00001), and the total effectiveness rate of the OCM-ET was 4.43 times that of the WM-A. The statistical heterogeneity among the studies was small (*P* > 0.05, *I*^2^ ≤ 50%) using the fixed-effects model. Analysis of subgroups based on the type of Chinese herbal decoctions used (classic or self-made) in the 16 studies indicated a significant difference in the total effectiveness rates between the two subgroups (self-made prescription: OR = 4.25, 95% CI = 2.34–7.71, *Z* = 4.76, *P* < 0.00001; Classic prescription: OR = 4.50, 95% CI = 3.07–6.61, *Z* = 7.68, and *P* < 0.00001). However, the statistical heterogeneity among the subgroups was small (*P* > 0.05, *I*^2^ ≤ 50%) (Figure 4).

**Figure 5:**
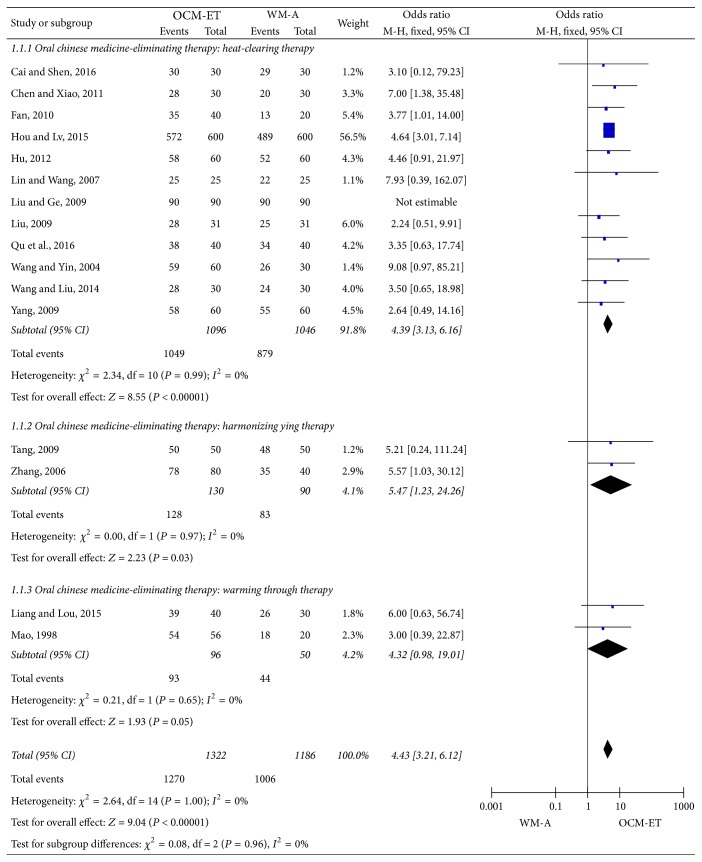
Meta-analysis of the total effectiveness rate against acute mastitis of Chinese oral medicine-Eliminating Therapy (OCM-ET) versus western medicine antibiotics (WM-A) based on the different therapeutic principles of the “Eliminating Therapy” used. CI indicates confidence interval.

Analysis of subgroups based on the therapeutic principle of “Eliminating Therapy” in the 16 studies also indicated a significant difference in the total effectiveness rates between the different subgroups (heat-clearing therapy: OR = 4.39, 95% CI = 3.13–6.16, *Z* = 8.55, and *P* < 0.00001; Harmonizing ying therapy: OR = 5.47, 95% CI = 1.23–24.26, *Z* = 2.23, *P* = 0.03; Warming through therapy: OR = 4.32, 95% CI = 0.98–19.01, *Z* = 1.93, *P* = 0.05). The warming through therapy and harmonizing ying therapy were in the margin of statistical significance, and further analysis is needed to expand the sample size. The statistical heterogeneity among the subgroups was small (*P* > 0.05, *I*^2^ ≤ 50%) (Figure 5).

### 3.5. Secondary Outcomes

**Figure 6:**
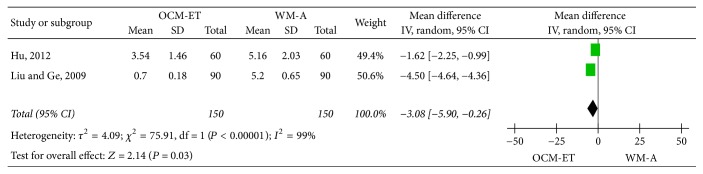
Meta-analysis of pain relief time in acute mastitis treated with Chinese oral medicine-Eliminating Therapy (OCM-ET) versus western medicine antibiotics (WM-A). CI indicates confidence interval.

*Pain Relief Time*. Two studies [23, 28] compared the time of pain relief (days) in OCM-ET and WM-A groups. The results of the meta-analysis using the random-effects model (*P* < 0.00001, *I*^2^ = 99%) indicated that the time taken to experience relief from pain was significantly shorter with OCM-ET than with WM-A. [MD = −3.08, 95% CI = (−5.90, −0.26), and *P* = 0.03] (Figure 6).

**Figure 7:**
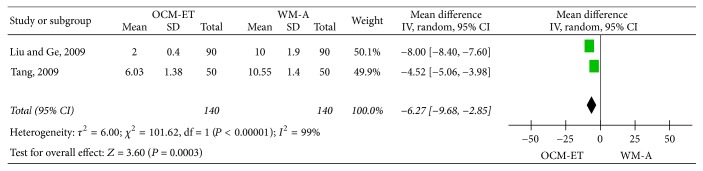
Meta-analysis of cure time in acute mastitis treated with Chinese oral medicine-Eliminating Therapy (OCM-ET) versus western medicine antibiotics (WM-A). CI indicates confidence interval.

*Cure Time*. Two studies [16, 23] compared the cure time (days) following treatment with OCM-ET and WM-A. The results of the meta-analysis using the random-effects model (*P* < 0.00001, *I*^2^ = 99%) indicated that OCM-ET had a significantly shorter cure time (MD = −6.27, 95% CI = (−9.68, −2.85), and *P* = 0.0003) (Figure 7).

### 3.6. Assessment of Publication Bias

**Figure 8:**
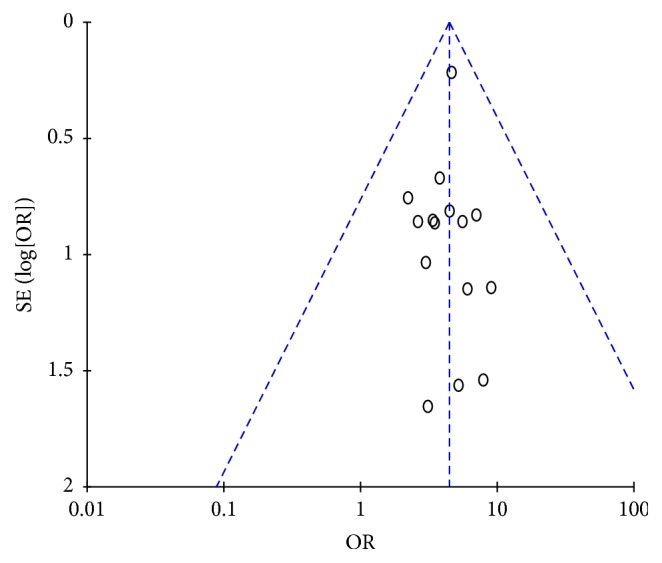
Funnel plot of comparison: Chinese oral medicine-Eliminating Therapy versus western medicine antibiotics treatment in acute mastitis.

In this review, the distribution of funnel plots was not completely symmetrical, probably due to a publication bias. This phenomenon may be related to the fact that studies with negative results or those that did not show statistical significance in their results were not published. Additionally, the bias could also be a result of the poor quality of methodologies used (Figure 8).

## 4. Discussion

TCM follows the therapeutic principle of “the therapy of elimination first,” which was first described by Wang Hongxu of the Qing Dynasty in the “Life-saving Manual of Diagnosis and Treatment of External Diseases.” The so-called “Eliminating Therapy” uses drugs that dissipate slowly to dissolve early stage sores, a method suitable for treating an abscess that has not yet become pus-filled. This systematic review mainly evaluates the curative effects of “Eliminating Therapy” reported in completely randomized controlled trials that used the Chinese medicinal techniques in the elimination method. These include relieving the exterior syndrome, purgative therapy, heat-clearing therapy, warming through therapy, phlegm removing therapy, dampness-eliminating therapy, qi-promoting therapy, and harmonizing ying therapy. These techniques can be used alone or in combination. A total of 16 papers were studied with a systematic assessment and meta-analysis to evaluate the curative effects quoted in the literature based on different techniques (self-prescription versus classical prescription and heat-clearing therapy versus warming through therapy versus harmonizing ying therapy) used in the elimination method. The literature included in this systematic review comprised mostly completely randomized controlled trials with small sample sizes and low methodological quality. Our meta-analysis showed that the effectiveness of the TCM and techniques in patients with early acute mastitis was significantly higher than that of western medicine (antibiotics). The TCM group also had significantly lower pain relief time and cure time compared to the western medicine group. Moreover, the differences between the subgroups were statistically significant, and the heterogeneity within each subgroup was less than when compared to the WM-A group. However, these findings need to be further analyzed using larger sample sizes. Furthermore, the antibiotics used in the included RCTs were not the standard drugs used in western medicine; future studies should also culture the breast milk and prescribe appropriate antibiotics when randomizing treatments.

The analysis of the literature revealed some problems. The quality scores for the trials were generally low. Even though all the chosen studies were based on the incorporation and elimination standards, most of them, with the exception of two studies [18, 28], did not describe the specific randomization method and distribution solutions used. As a result, there is a high possibility that the implementation, measurement, and selection were all biased. Moreover, none of the 16 articles describe whether a blind method was used for the traditional Chinese medicinal broth. The funnel figure also shows an incomplete distribution, suggesting a possible publication bias. Therefore, the results of this study have to be confirmed by more rigorous multicenter, randomized, and double-blinded clinic trials with larger sample sizes. Acute mastitis is characterized by recurrence, which should be considered when evaluating the curative effect. Most of the studies that were evaluated in the study only reported the short-term results and the overall efficacy of the treatment for the initial stages of acute mastitis and not the followup studies and long-term results. Only one study [16] reported the rate of recurrence, which may cause a certain bias in the results. Similarly, only one study [19] reported adverse reactions such as diarrhea within 2 days of taking the medicine, which disappeared once the treatment was stopped.

Acute mastitis is an inflammatory condition of the lactiferous ducts and the surrounding connective tissue. It starts with stagnation of breast milk (the stagnant stage). According to TCM, postpartum qi or blood deficiency, or both lead to a cold and damp stasis in the carbuncle. Lactation lasts for many months, and if women with breast milk deposition fail to receive treatment on time or receive the wrong treatment, the disease could take a longer course or keep recurring [13]. The meta-analysis results confirmed the curative effects of elimination oral Chinese medicine treatment for the initial stages of acute mastitis. While the trials evaluated in this study used different types of oral Chinese medicines, such as Trichosanthes burdock soup, disinfectant soup, YangHe decoctions, and self-developed TCM prescriptions, the therapeutic principles of elimination are commonly used to eliminate blockages in patients with initial stage acute mastitis. Heat-clearing therapy, warming through therapy, and ying-harmonizing therapy all belong to the category of “elimination” in TCM. However, there are no objective criteria for the ingredients and dosages of TCM prescriptions. This study evaluated the treatment of acute mastitis in its initial stages, following the principle of “elimination therapy,” in order to determine the TCMs that are stable and more effective, with the goal of promoting the modernization of TCM.

## 5. Conclusions

While the evidence that OCM-ET may be an effective treatment for early acute mastitis is encouraging, it is not conclusive owing to the low methodological quality of the RCTs and the lack of use of standard antibiotics the studies. Therefore, high-quality RCTs, with low risk of bias and adequate sample sizes, are required to confirm the findings of this study.

## Figures and Tables

**Table 1:** Included RCTs.

Study	Location	baseline	Age (years) E/C	Duration (days) E/C	Duration of treatment (days)	Sample size E/C	Staging of the acute mastitis	ADs of experimental group	FUP (months)
Liu and Ge, 2009	China	Comparable	27.2/24.5	3.5/3.3	NR	90/90	Galactostasis stage	NR	NR
Chen and Xiao, 2011	China	Comparable	29/29.5	3/2.5	7	30/30	Galactostasis stage	NR	NR
Zhang, 2006	China	NR	NR	NR	7	80/40	Galactostasis stage	NR	NR
Wang and Yin, 2004	China	NR	NR	NR	7	60/30	Galactostasis stage	NR	NR
Wang and Liu, 2014	China	Comparable	28.5/29.5	5.5/5.5	NR	30/30	Galactostasis stage	NR	NR
Yang, 2009	China	Comparable	28/29	NR	7	60/60	Galactostasis stage	NR	NR
Liang and Lou, 2015	China	Comparable	26.68/26.98	13.98/14.28	14	40/30/30	Galactostasis stage	NR	NR
Liu, 2009	China	Comparable	27.6/27.2	4.6/4.5	3	31/31	Galactostasis stage	NR	NR
Tang, 2009	China	Comparable	25.23/26.56	4.5/3.98	7	50/50	Galactostasis stage	NR	1–3
Hu, 2012	China	Comparable	27.45/27.45	4.02/4.02	7	60/60	Galactostasis stage	NR	NR
Fan, 2010	China	NR	28/28	8.5/8.5	4	40/20	Galactostasis stage	NR	NR
Lin and Wang, 2007	China	Comparable	28.5/28	NR	3	25/25	Galactostasis stage	NR	NR
Hou and Lv, 2015	China	Comparable	33.9/35.7	24.6/25.9	NR	600/600	Galactostasis stage	NR	NR
Mao, 1998	China	Comparable	25/25	NR	NR	56/20	Galactostasis stage	NR	NR
Cai and Shen, 2016	China	Comparable	30.30/29.97	2.03/1.93	6	30/30	Galactostasis stage	One patient: diarrhea	NR
Qu et al., 2016	China	Comparable	28/28	3.5	7	40/40	Galactostasis stage	NR	NR

RCTs: randomized controlled trials; E: experimental group; C: control group; ADs: adverse events; FUP: followup period; NR: no report.

**Table 2:** Treatments used in the included studies.

Study	Interventions		Suppliers of the TCM	Therapeutic principle of the TCM	Main outcomes	Evaluation methods
	Experimental group	Control group				
Liu and Ge, 2009	Self-made mammary abscess eliminating carbuncle decoction	Penicillin	The Third Affiliated Hospital of Luohe Medical College	Heat-clearing and detoxifying, eliminating carbuncle, and resolving masses	Curative rate, pain relief time, mass reduction time, and cure time	Clinical symptoms and physical sign, mass integral
Chen and Xiao, 2011	GuaLouNiuBang decoction	Cefadroxil or ceftriaxone	Xiamen traditional Chinese Medicine Hospital of Fujian Province	Relieving the depressed liver, heat-clearing, and resolving masses	Total effective rate, blood routine	The integral of clinical symptom and physical sign
Zhang, 2006	HuaHong eliminating carbuncle powder	Penicillin + ampicillin	Shandong Zouping hospital of Traditional Chinese Medicine	Clots absorption and dredging collaterals, eliminating carbuncle, and resolving masses	Total effective rate, blood routine	Clinical symptoms and physical sign, mass integral
Wang and Yin, 2004	Self-made detoxification and resolving masses decoction	Penicillin + ampicillin	Binzhou institution hospital	Heat-clearing and detoxifying, soothing liver, and regulating stomach	Total effective rate, blood routine	Clinical symptoms and physical sign, mass integral
Wang and Liu, 2014	Self-made mammary abscess formula 1	Cefoxitin	Tianjin Hospital of Integrated Traditional Chinese and Western Medicine	Heat-clearing and detoxifying, soothing liver, and lactogenesis	Total effective rate, blood routine	Clinical symptoms and physical sign, mass integral
Yang, 2009	Soothing liver and lactogenesis decoction	Cefuroxime	Shanghai Hospital of Traditional Chinese Medicine	Soothing liver and clearing stomach, lactogenesis, and resolving masses	Total effective rate, blood routine, duration of treatment, and lactation situation	Clinical symptoms and physical sign, mass integral
Liang and Lou, 2015	YangHe decoction	Penicillin	Zhejiang General Hospital, Zhejiang University of Traditional Chinese Medicine	Warming Yang, and dredging collaterals, and resolving hard lump	Total effective rate, curative rate, WBC, neutrophils percentage, and CRP	Lou's assessment quantitative integral tables of the curative effect for breast carbuncle
Liu, 2009	Disinfectant soup	Cefoperazone sulbactam	Beijing Electrical Hospital	Heat-clearing and detoxifying, lactogenesis, and eliminating carbuncle	Total effective rate, OR, and NNT	Clinical symptoms and physical sign, mass integral
Tang, 2009	ZhiDanxiaoru decoction	Cefotaxime	Hebei Huailai Hospital of Traditional Chinese Medicine	Eliminating heat, purging fire, cooling blood, and detumescence	Total effective rate, curative rate, recurrence rate, cure, and effective time required for treatment	Clinical symptoms and physical sign, mass integral
Hu, 2012	Modified herbal decoction	Penicillin	Zhejiang Yongkang Maternity and Child Care Hospital	Heat-clearing and detoxifying, resolving masses, and dredging collaterals	Total effective rate, recovery time for temperature, and pain relief time	Clinical symptoms and physical sign, mass integral
Fan, 2010	Self-made PuXia decoction	Penicillin	The Second People's Hospital of Kunshan	Heat-clearing and detoxifying, detumescence, and resolving masses	Total effective rate	Clinical symptoms and physical sign, mass integral
Lin and Wang, 2007	Single Taraxacum decoction	Cephradine	Qingdao Central Hospital	Heat-clearing and detoxifying, antiphlogistic, and clots absorption	Total effective rate, average effective time	Clinical symptoms and physical sign, mass integral
Hou and Lv, 2015	GuaLouNiuBang decoction	Cefazolin pentahydrate	Chongqing Hospital of Traditional Chinese Medicine	Detumescence and lactogenesis, soothing liver, and clearing stomach	Total effective rate	Clinical symptoms and physical sign, mass integral
Mao, 1998	YangHe decoction	Cephradine	Zhejiang Hangzhou Gongshu Hospital of Integrated Traditional Chinese and Western Medicine	Warming Yang and dredging collaterals	Total effective rate	Mass integral
Cai and Shen, 2016	GuaLouNiuBang decoction	Penicillin	Minhang Branch of Yueyang Hospital of Integrated Traditional Chinese and Western Medicine, Shanghai University of Traditional Chinese Medicine	Heat-clearing and detoxifying, lactogenesis, and resolving masses	Total effective rate, fever clearance time, pain relief time, and resolving mass time	Clinical symptoms and physical sign, mass integral
Qu et al., 2016	Self-made lactogenesis and resolving masses decoction	Penicillin	Zhejiang Lishui hospital of Traditional Chinese Medicine	Heat-clearing and detoxifying, resolving masses, and Eliminating carbuncle	Total effective rate	Clinical symptoms and physical sign, mass integral

WBC: white blood cell; CRP: C-reactive protein; OR: odds ratio; NNT: number needed to treat.
